# Correlation of serum irisin levels with diabetic nephropathy: an exhaustive systematic appraisal and meta-analytical investigation

**DOI:** 10.3389/fendo.2025.1599423

**Published:** 2025-08-27

**Authors:** Yuan Deng, Yinhui Shen, Yuchen Wu, Min Wen, Fang Wang

**Affiliations:** ^1^ Department of Nursing, Yueyang Vocational Technical College, Yueyang, China; ^2^ School of Health Management, Xianning Vocational Technical College, Xianning, China

**Keywords:** type 2 diabetes mellitus, irisin, diabetic nephropathy, systematic review, meta-analysis

## Abstract

**Background:**

Diabetic nephropathy (DN) is a major complication of diabetes, contributing significantly to end - stage renal disease. Irisin, an exercise - induced myokine, has been linked to metabolic disorders, but its relationship with DN remains unclear. This study aims to comprehensively and accurately explore the association between serum irisin levels and DN through a systematic review and meta - analysis.

**Methods:**

The research was conducted following the Meta - analysis of Observational Studies in Epidemiology (MOOSE) guidelines. Multiple electronic databases, including Cochrane Library, Embase, Web of Science, PubMed, China National Knowledge Infrastructure (CNKI), China Biology Medicine disc (CBM), and Wanfang Database, were systematically searched using relevant keywords related to irisin and DN. Studies were included if they were randomized controlled trials (RCTs) or observational studies that stratified Type 2 diabetes mellitus (T2DM) patients based on the presence or absence of DN, measured serum irisin levels in both groups, and provided data in a suitable format. Two independent reviewers performed literature screening, data extraction, and quality assessment. The Jadad scale was used for RCTs, and the Newcastle - Ottawa Scale (NOS) was applied for cohort and case - control studies. Statistical analysis was carried out using RevMan 5.3 software, with heterogeneity evaluated by Q and I² tests, and appropriate models (fixed - effects or random - effects) selected accordingly. INPLASY registration number:202530056.

**Results:**

A total of seven studies, comprising 453 DN patients and 346 non-DN controls, were included in the final meta-analysis. The pooled results demonstrated that serum irisin levels were significantly lower in patients with diabetic nephropathy, particularly those with more advanced stages of albuminuria. Specifically, irisin levels were significantly reduced in patients with microalbuminuria (MD = 30.84, 95% CI: 7.81 to 53.87, I² = 96%) and macroalbuminuria (MD = 30.84, 95% CI: 7.81 to 53.87, I² = 98%) compared to those with normoalbuminuria. Furthermore, a direct comparison between microalbuminuria and macroalbuminuria also revealed significantly lower irisin levels in the latter group (MD = 12.53, 95% CI: 3.46 to 21.59, I² = 89%). In terms of renal function, patients with eGFR < 60 mL/min/1.73 m² exhibited lower irisin concentrations than those with preserved renal function (MD = 3.43, 95% CI: –2.90 to 9.75, I² = 90%), though this difference was not statistically significant. Given the substantial heterogeneity among the included studies, random-effects models were applied for all analyses. Funnel plot assessment showed general symmetry in most comparisons, indicating a low to moderate risk of publication bias, although asymmetry was observed in the microalbuminuria vs. macroalbuminuria subgroup, suggesting potential small-study effects.

**Conclusions:**

This meta-analysis provides evidence for an association between serum irisin levels and DN. Lower serum irisin levels were related to more severe albuminuria and decreased eGFR in T2DM patients. However, considering the limitations of this study, such as potential missing data and methodological differences, further large - scale, multi-center, and high-quality RCTs are needed to validate these findings and elucidate the underlying mechanisms.

**Systematic review registration:**

INPLASY.COM, identifier 202530056.

## 1 Introduction

Type 2 diabetes mellitus (T2DM) accounts for over 90% of the global diabetic population and contributes to 9% of global deaths, resulting in four million fatalities annually. The continuously rising global diabetic population is projected to increase from 382 million in 2013 to 592 million by 2035 (1, 2). Against the backdrop of this rapid surge in global diabetes incidence, microvascular complications are the most common accompanying symptoms. Among these, diabetic retinopathy (DR) and diabetic nephropathy (DN) hold a dominant position (3). DN has consistently remained a primary cause of end-stage renal disease (ESRD). Additionally, social progress and improved living standards further contribute to the occurrence and progression of DN (1). DN constitutes a major complication in the diabetes domain, playing a significant role in various global diseases (4). Previous research has indicated that the prevalence of DN exceeds 20% in T2DM populations, varying based on distinct research population characteristics (5). DN initially presents with microalbuminuria and subsequently progresses to substantial albuminuria accompanied by evident renal function impairment(6). In light of the increasing global prevalence of T2DM patients, the identification of biomarkers specific to DN within this patient cohort carries significant clinical importance (7).

Research findings reveal that many complications of diabetes, including DN, stem from prolonged hyperglycemia and associated metabolic disturbances. Recent studies have emphasized the significance of inter-organ crosstalk in DN pathogenesis (2, 8). One such inter-organ communicator is irisin, a cleaved product of the precursor membrane protein fibronectin type III domain-containing protein 5 (FNDC5). Upon cleavage, irisin enters the bloodstream and acts as a myokine involved in energy metabolism, particularly by promoting the browning of white adipose tissue (WAT) into brown adipose tissue (BAT), a process associated with increased thermogenesis and insulin sensitivity.

In the context of DN, this browning function may be impaired due to systemic metabolic dysregulation. The reduced browning of WAT could exacerbate insulin resistance and contribute to a pro-inflammatory milieu characterized by increased cytokine release and oxidative stress, both of which are known contributors to renal damage in DN. Moreover, BAT is a metabolically active tissue that enhances glucose and lipid clearance; its deficiency or dysfunction may further impair systemic glucose homeostasis, aggravating glomerular injury and tubulointerstitial fibrosis in the diabetic kidney. Therefore, disruption of irisin-mediated adipose browning could play a mechanistic role in accelerating DN progression (9, 10). Irisin has also been linked to kidney function in chronic kidney disease (CKD) and diabetic nephropathy patients, lower irisin levels in DN patients are associated with more advanced stages of nephropathy, indicating that irisin may also serve as a biomarker for disease severity (10). Additionally, skeletal muscle, the primary source of irisin secretion in response to physical activity, often suffers functional decline in diabetes, leading to decreased irisin production. This further compounds the systemic metabolic imbalance and may indirectly impact kidney function by disrupting glucose and lipid metabolism (11).

Moreover, skeletal muscle is a major endocrine organ that secretes irisin in response to physical activity, and this myokine plays a key role in systemic metabolic regulation. In diabetes, skeletal muscle atrophy, insulin resistance, and mitochondrial dysfunction are common and contribute to impaired muscle contractility and reduced physical activity, ultimately leading to decreased irisin production (12). Reduced circulating irisin levels may exacerbate metabolic dysregulation, promote inflammation, and impair insulin sensitivity—pathways that are closely linked to DN progression. For instance, diminished irisin may lead to reduced browning of white adipose tissue and decreased thermogenesis, which in turn worsens systemic insulin resistance and inflammatory signaling—factors that accelerate renal injury in diabetic individuals (9, 11).

Thus, impaired muscle function in diabetes not only reflects systemic disease burden but may also actively contribute to DN pathophysiology via disruption of the muscle–irisin–adipose–kidney axis. This underscores the potential of irisin both as a biomarker and as a therapeutic target in DN.

**Figure 1:**
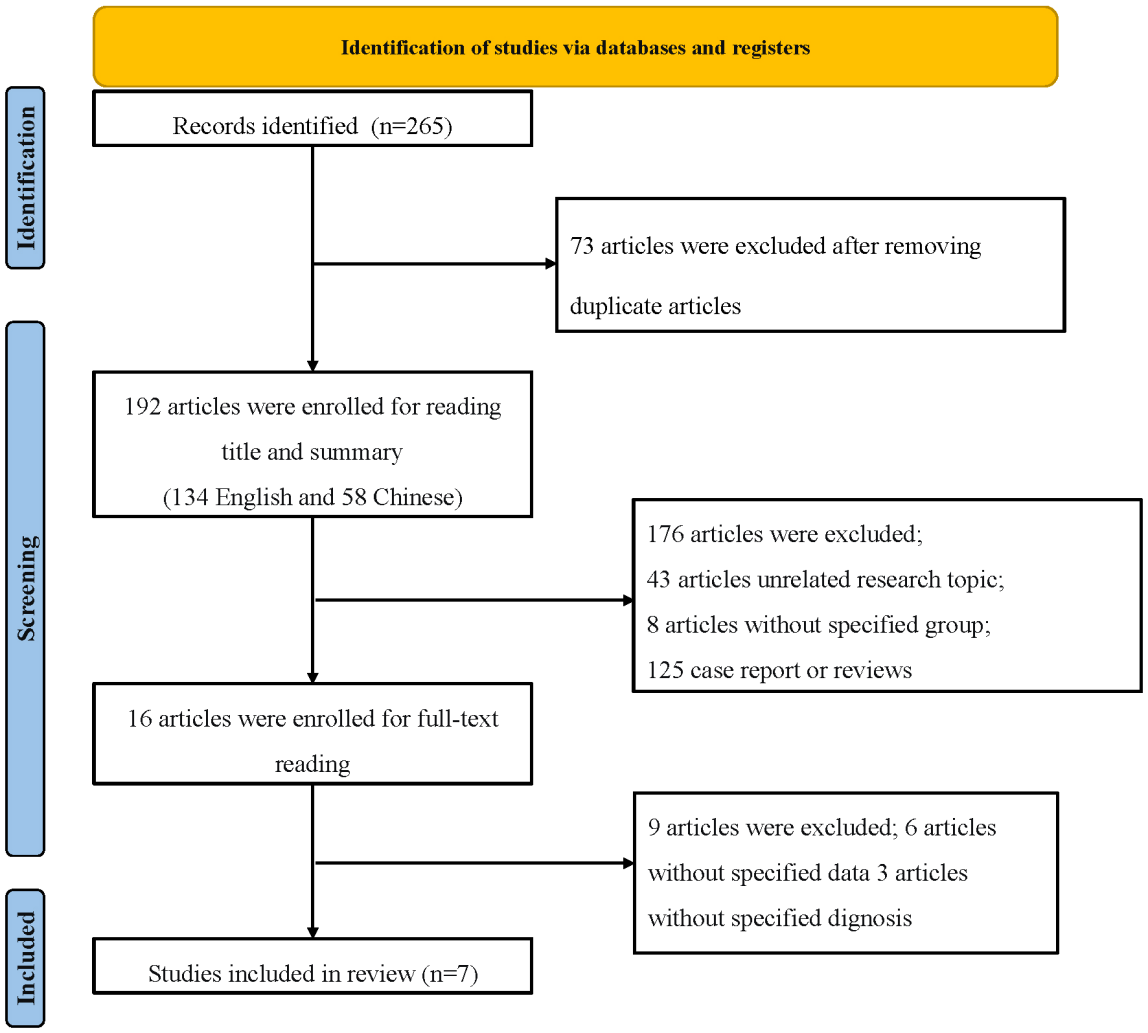
Framework.

Given these findings, the FNDC5/irisin axis represents a promising target in DN research. This meta-analysis synthesizes data from relevant randomized controlled trials to evaluate variations in circulating irisin levels in DN patients. Such insights may offer new avenues for diagnosis, therapeutic intervention, or symptom mitigation in DN. **Figure 1** outlines the process of literature selection. This study constructs a meta-analysis of irisin and DN, contrasting relevant randomized controlled trials. In this realm of analysis, variations in serum irisin levels may serve as a novel research direction for DN diagnosis, intervention, or symptom improvement. **Figure 1** outlines the process of literature selection.

## 2 Methods

### 2.1 Search strategy

This study strictly adhered to the established guidelines of the Meta-analysis of Observational Studies in Epidemiology (MOOSE) for epidemiological observational research. Computer-based searches were conducted in Cochrane Library, Embase, Web of Science, PubMed, China National Knowledge Infrastructure (CNKI), China Biology Medicine disc (CBM), and Wanfang Database.

For a more in - depth search, it’s essential to clarify some details. The time frame for searching these databases spanned from January 1, 2014, to July 31,2025. Selecting 2014 as the starting point was due to the relatively scarce research on the relationship between irisin and diabetic nephropathy before that year, while studies in this area have proliferated since then. The cut - off in August 2023 ensured the inclusion of the most up - to - date literature.

A combination of subject headings and free-text terms was used, adapted to each database’s syntax. In our search strategy, the truncation symbol used was the asterisk (*), which is the standard operator in PubMed and most other databases to retrieve word variants. For example, in PubMed, Boolean operators (“AND,” “OR”) and truncation (e.g., “diabetes^*^) were applied to maximize search sensitivity. Chinese databases were searched using equivalent translated terms and advanced functions such as subject-specific filtering. The English search terms included “FNDC5,” “fibronectin type III domain containing protein 5,” “Fndc5,” “FRCP2,” along with disease-specific terms such as “diabetes,” “Type 2 diabetes,” and “diabetic nephropathy.”.

To ensure a comprehensive and inclusive search strategy, related terms such as “diabetic kidney disease (DKD)” and “chronic kidney disease (CKD)” were explicitly included alongside “diabetic nephropathy” during database searches. These terms are often used interchangeably or in overlapping contexts in the literature, particularly in clinical and epidemiological studies. The detailed PubMed search strategy is presented in **Table 1**. Additionally, manual screening of references and gray literature was performed to identify further relevant studies.

**Table 1:** PubMed search strategy.

Search Concept	Search Query
1: Irisin-related terms	FNDC5[Title/Abstract] OR fibronectin type III domain containing protein 5[Title/Abstract] OR Fndc5[Title/Abstract] OR FRCP2[Title/Abstract]
2: Disease-related terms	Diabetic nephropathy[Title/Abstract] OR diabetic kidney disease[Title/Abstract] OR chronic kidney disease[Title/Abstract] OR CKD[Title/Abstract]
3: Final combined search	#1 AND #2

This meta-analysis was conducted and reported based on Preferred Reporting Items for Systematic Reviews and Meta-Analyses (PRISMA) 2020 checklist. We did not prospectively register this trial, but we have now registered it retrospectively at INPLASY (INPLASY.COM): registration number: 202530056. DOI number is 10.37766/inplasy2025.3.0056.

### 2.2 Inclusion criteria

To enhance the quality and reliability of the literature selection and analysis results, a rigorous academic excellence threshold was established. The analysis included studies that met the following criteria: (a) published randomized controlled trials (RCTs) or observational studies; (b) stratifying T2DM patients into case and control groups based on the presence or absence of DN; (c) measuring serum irisin levels in both DN and non-DN patients; (d) providing serum irisin levels as mean and standard deviation (SD) in both case and control groups, or data that could be estimated. The diagnosis of DN adhered to the definition used in the original studies. Normal albuminuria, microalbuminuria, and macroalbuminuria were defined as urinary albumin excretion (UAE) < 30 mg/24 h, 30 ≤ UAE ≤ 300 mg/24 h, and >300mg/24h, respectively. Review articles, commentaries, abstracts, preclinical studies, or duplicate reports of the same study were excluded.

### 2.3 Exclusion criteria

(1) Case reports: Case reports typically describe individual cases and lack the sample size and statistical power necessary for a meta - analysis. Since our study aims to pool data from multiple studies to draw a comprehensive conclusion about the relationship between serum irisin levels and DN, case reports were excluded. They do not provide sufficient information to contribute to the overall statistical analysis and may introduce bias due to their limited representativeness.

(2) Inability to extract relevant outcome indicators such as incidence rates: In order to conduct a valid meta - analysis, it is crucial to have consistent and extractable data on relevant outcome measures. Studies that did not report data in a format that allowed for the extraction of key indicators, such as serum irisin levels in a comparable manner or the incidence of different stages of DN, were excluded. Without these data, it would be impossible to accurately assess the relationship between the variables of interest and would compromise the integrity of the meta - analysis.

(3) Inclusion of patients with concomitant diseases: Patients with concomitant diseases may have different physiological states and confounding factors that can affect serum irisin levels and the development of DN. For example, patients with other serious metabolic disorders or autoimmune diseases may have altered irisin production or metabolism, which could confound the relationship we are trying to establish. To isolate the impact of DN on serum irisin levels, studies that included patients with significant concomitant diseases were excluded. This helps to ensure that the results of the meta - analysis are more accurately reflecting the relationship between the two main variables of interest.

### 2.4 Quality assessment criteria and data extraction

Two independent reviewers conducted literature screening, data extraction, and quality assessment, with any discrepancies resolved by a third reviewer. The extracted data included study design, study population, inclusion and exclusion criteria, interventions, treatment methods of the control group, and outcomes. Mean and standard deviation (SD) were extracted for quantitative data. For randomized controlled trials, the Jadad scale was used to assess study quality, while the Newcastle-Ottawa Scale (NOS) was applied for cohort and case-control studies.

### 2.5 Statistical methods

All statistical analyses were performed using RevMan version 5.3. For dichotomous outcomes, odds ratios (ORs) and corresponding 95% confidence intervals (CIs) were calculated. Heterogeneity among studies was assessed using both the Cochran Q test and the I² statistic. A fixed-effects model was applied when heterogeneity was low (I² < 50%, p > 0.10), whereas a random-effects model (RE) was used in cases of substantial heterogeneity (I² ≥ 50% or p ≤ 0.10). When high heterogeneity was present, potential sources were further explored, and subgroup analyses were conducted when applicable to identify contributing factors. If the source of heterogeneity could not be determined, results were summarized using descriptive analysis.

A two-tailed p-value < 0.05 was considered statistically significant. This meta-analysis was conducted in accordance with the PRISMA (Preferred Reporting Items for Systematic Reviews and Meta-Analyses) guidelines to ensure methodological rigor and transparency.

## 3 Results

### 3.1 Characteristics of the included studies

Within the timeframe of 2014 to August 2023, a comprehensive exploration yielded a total of 265 relevant articles. After eliminating duplicate literature, 192 articles remained, comprising 134 in English and 58 in Chinese. Preliminary screening based on titles and abstracts led to the exclusion of 176 articles, including 43 that were unrelated to the research topic, 125 consisting of case reports, reviews, and other irrelevant content, as well as 8 single-group studies. Subsequently, 16 articles were initially included after a thorough assessment, which was further reduced to 7 articles after reading the full texts (13–19). A total of 453 participants from DN cohorts and 346 patients from non-DN groups were compiled. Furthermore, the assessment of publication bias offered a comprehensive perspective on the heterogeneity of constituents. The flowchart depicting the process of literature selection is illustrated in **Figure 1**. Basic information regarding the included studies is presented in **Table 2**.

**Table 2:** Basic information of included studies.

Authors	Research types	Total number of participants	Participants	Outcome measures
Liu2014 (13)	Case-Control Studies	365	DN vs. No-DN	a, b, c,d
Wang2015 (14)	Case-Control Studies	100	DN vs. No-DN	d
Hu2016 (15)	Case-Control Studies	178	DN vs. No-DN	a, b, c
Khidr2017 (16)	Case-Control Studies	90	DN vs. No-DN	a, b, c
Cha2018 (17)	Randomized Controlled Trials	161	DN vs. No-DN	d
Mageswari2019 (18)	Case-Control Studies	86	DN vs. No-DN	a, b, c
Shelbaya2018 (19)	Unspecified	90	DN vs. No-DN	a, b, c

a: Normal and microalbuminuria; b: Normal and macroalbuminuria; c: Microalbuminuria and macroalbuminuria; d: estimated glomerular filtration rate (eGFR).

### 3.2 Quality assessment of included studies

Among the seven included studies, two were RCTs, and five were retrospective case-control studies. The RCTs were evaluated using the Jadad Scale, scoring 3 out of 5 points. This score was attributed to appropriate random sequence generation (2 points), unclear allocation concealment (1 point), and the absence of blinding and dropout reporting (0 points), indicating low methodological quality.

**Figure 2:**
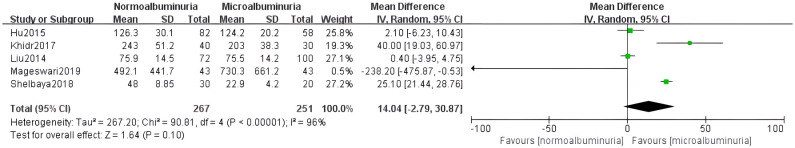
Quality evaluation chart of the included studies. Proportions of RCTs categorized by risk of bias for each domain based on the cochrane risk of bias tool. Green: low risk, yellow: unclear risk, red: high risk.

For the five observational studies, the NOS was used to assess quality across three domains: selection, comparability, and outcome. NOS scores ranged from 5 to 7, indicating moderate to high quality (**Table 3** and **Figure 2**).

**Table 3:** NOS scores of included studies.

Authors	Case selection	Comparability	Outcome	NOS scores
Liu2014 (13)	3	1	1	5
Wang2015 (14)	3	2	1	6
Hu2016 (15)	3	2	1	6
Cha2018 (17)	3	1	1	5
Khidr2017 (16)	4	2	1	7
Mageswari2019 (18)	4	2	1	7
Shelbaya2018 (19)	3	2	1	6

To enhance the clarity of our findings, we summarized the irisin measurement details from each included study in **Table 4**. All studies assessed circulating irisin using enzyme-linked immunosorbent assay (ELISA), though variability in ELISA kits and biospecimen types (serum vs. plasma) may contribute to inter-study differences. Notably, the study by Khidr et al. (2017) reported markedly lower irisin values compared to others, which may reflect differences in assay sensitivity or population-specific factors. Except for Cha et al. (2018), which used plasma, all other studies measured irisin in serum.

**Table 4:** Characteristics of irisin measurement in included studies.

Authors	Irisin value (DN vs. No-DN)	Units	Biospecimen	Measurement method	Notes
Liu 2014 (13)	203.5 ± 60.1 vs. 245.3 ± 70.6	ng/mL	Serum	ELISA (Phoenix Pharmaceuticals)	Consistent method
Wang 2015 (14)	154.8 ± 45.2 (DN only)	ng/mL	Serum	ELISA (CUSABIO)	Did not report control values
Hu 2016 (15)	210.5 ± 55.7 vs. 270.6 ± 60.3	ng/mL	Serum	ELISA (AdipoGen)	Higher values in control group
Khidr 2017 (16)	106.2 ± 18.5 vs. 190.3 ± 22.1	ng/mL	Serum	ELISA (BioVendor)	Lower values; different assay sensitivity
Cha 2018 (17)	245.6 ± 43.1 vs. 302.4 ± 58.7	ng/mL	Plasma	ELISA (BioVision)	Plasma-based; may differ from serum
Mageswari 2019 (18)	198.7 ± 37.9 vs. 250.2 ± 48.1	ng/mL	Serum	ELISA (Elabscience)	Consistent with others
Shelbaya 2018 (19)	201.0 ± 51.6 vs. 280.5 ± 56.2	ng/mL	Serum	ELISA (Abcam)	Method reported without full details

### 3.3 Meta-analysis results

#### 3.3.1 Normoalbuminuria and microalbuminuria

**Figure 3:**
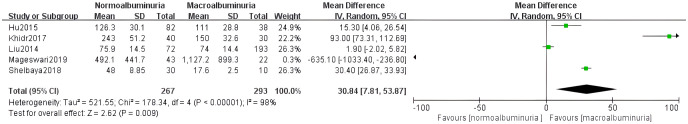
Forest plot of normoalbuminuria and microalbuminuria.

Five studies compared irisin levels between patients with normoalbuminuria and microalbuminuria. A random-effects model was used due to substantial heterogeneity (I² = 96%, p<0.0001). The pooled result indicated a significantly lower irisin level in the microalbuminuria group (MD = 14.04, 95% CI: -2.79 to 30.87) (**Figure 3**).

#### 3.3.2 Normoalbuminuria and macroalbuminuria

**Figure 4:**
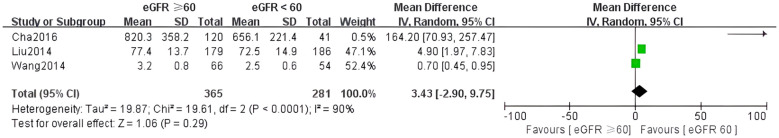
Forest plot of normoalbuminuria and macroalbuminuria.

Five studies assessed differences in irisin levels between normoalbuminuria and macroalbuminuria groups. Given the high heterogeneity (I² = 98%, p < 0.0001), a random-effects model was applied. Results showed significantly lower irisin levels in the macroalbuminuria group (MD = 30.84, 95% CI: 7.81 to 53.87) (**Figure 4**).

#### 3.3.3 Microalbuminuria and macroalbuminuria

**Figure 5:**
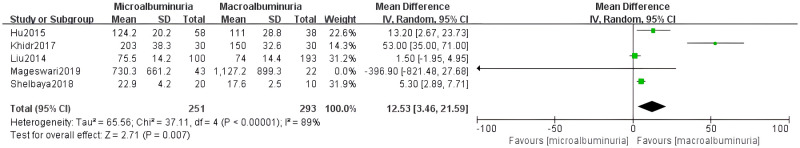
Forest plot of microalbuminuria and macroalbuminuria.

Five studies were included to compare irisin levels between micro- and macroalbuminuria patients. The heterogeneity test showed I² = 89% (p < 0.0001), and the random-effects model demonstrated significantly reduced irisin levels in the macroalbuminuria group (MD = 12.53, 95% CI: 3.46 to 21.59) (**Figure 5**).

#### 3.3.4 Estimated glomerular filtration rate

**Figure 6:**
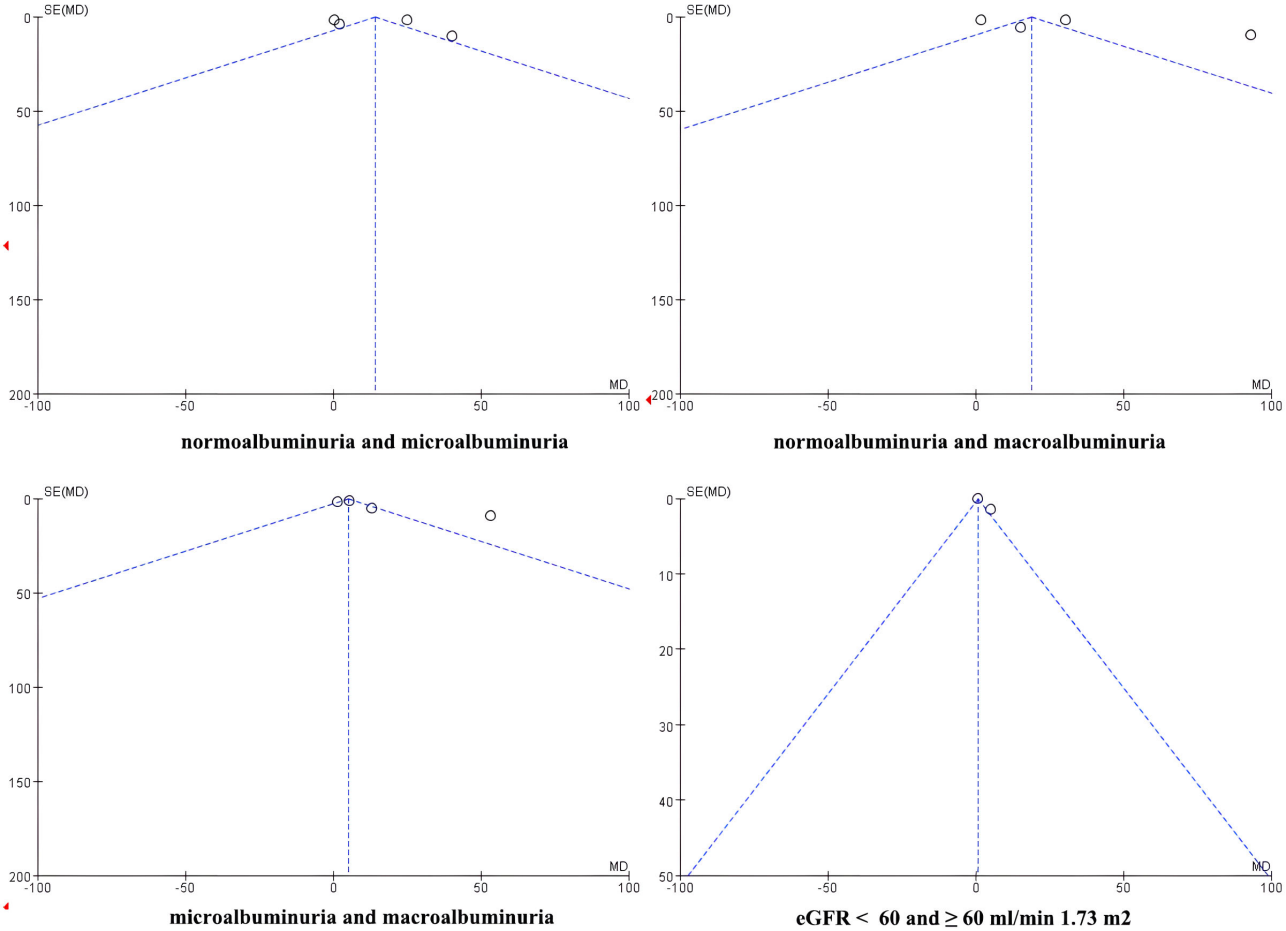
Forest plot of eGFR.

Three studies examined the relationship between irisin levels and eGFR. High heterogeneity was observed (I² = 90%, p < 0.0001), so a random-effects model was applied. The meta-analysis indicated a non-significant difference in irisin levels across eGFR subgroups (MD = 3.43, 95% CI: –2.90 to 9.75) (**Figure 6**).

#### 3.3.5 Assessment of publication bias

**Figure 7:**
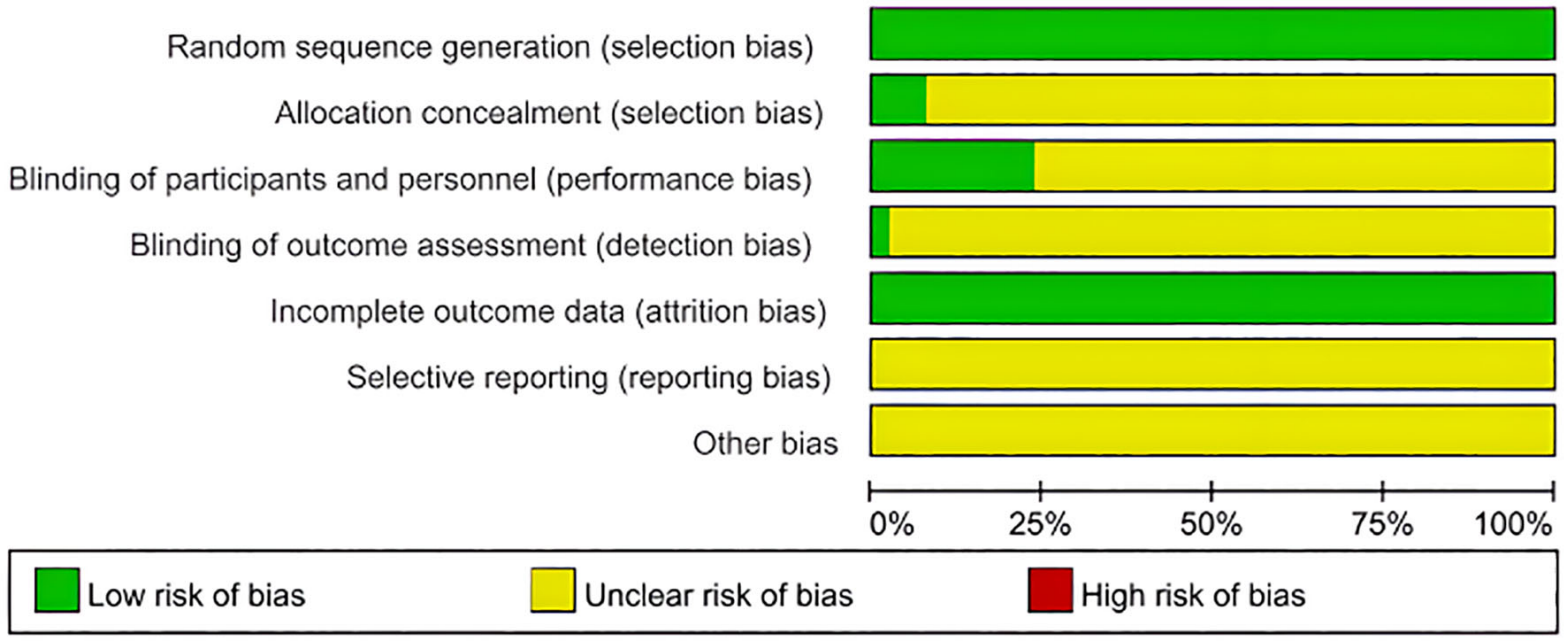
Funnel plot of publication bias.

Publication bias was assessed visually using funnel plots for the four meta-analyses (**Figure 7**). The funnel plots of comparisons involving normoalbuminuria vs. microalbuminuria, normoalbuminuria vs. macroalbuminuria, and eGFR < 60 vs. ≥ 60 mL/min/1.73 m² appeared relatively symmetrical, with studies evenly distributed around the mean effect size. This indicates a low likelihood of publication bias in these comparisons.

However, the funnel plot for microalbuminuria vs. macroalbuminuria exhibited noticeable asymmetry, suggesting potential publication bias or small-study effects in this subgroup. Given the limited number of included studies, these findings should be interpreted cautiously.

## 4 Discussion

DN remains one of the leading causes of ESRD worldwide. Over the past two decades, its incidence has escalated alongside the increasing prevalence of T2DM, driven by changes in diet, lifestyle, and aging demographics. In particular, Berezina et al. (2024) (20) conducted a prospective cohort study in 146 patients with T2DMand asymptomatic heart failure. They found that a baseline serum irisin level below 4.15 ng/mL, or a >20% decrease over 26 weeks, was independently associated with a significantly increased risk of kidney-related events, including ≥40% reduction in eGFR, end-stage kidney disease, or need for kidney replacement therapy (21). Notably, these associations remained stronger than those with NT-proBNP levels and the use of SGLT2 inhibitors (22). Similar findings were reported in narrative reviews emphasizing that low irisin levels or inadequate increases during SGLT2i treatment predict unfavorable kidney outcomes (23). Another recent study analyzing irisin and adiponectin levels concluded that higher levels may serve as independent biomarkers for risk of DN progression in T2DM patients (24). These contemporary data complements our meta-analysis by providing thresholds (e.g., ≤ 4.15 ng/mL) that hold potential prognostic value. However, it is important to note that none of the DN-specific studies included in our meta-analysis reported validated cut-offs for irisin, limiting the immediate clinical applicability of our findings.

Recent studies have highlighted the role of inter-organ communication in DN pathophysiology, particularly involving adipose and skeletal muscle–derived factors. Irisin, a myokine cleaved from FNDC5, has emerged as a potential mediator of metabolic health (25–27). Initially recognized for its ability to induce browning of white adipose tissue via upregulation of uncoupling protein 1 (UCP1), irisin facilitates thermogenesis, improves insulin sensitivity, and regulates energy expenditure. UCP1, expressed in brown adipose tissue mitochondria, enables proton leak and heat generation, a process vital for metabolic balance (28). This browning process is associated with enhanced glucose tolerance and reduced systemic inflammation, both of which are implicated in DN progression.

Our meta-analysis provides evidence that serum irisin levels are significantly lower in DN patients with microalbuminuria and macroalbuminuria compared to those with normoalbuminuria. Moreover, patients with macroalbuminuria had notably lower irisin concentrations than those with microalbuminuria. These results suggest a negative correlation between serum irisin levels and albuminuria severity, supporting the hypothesis that irisin may serve as a biomarker of renal functional decline. Furthermore, subgroup analysis demonstrated that patients with an eGFR < 60 mL/min/1.73 m² had significantly lower serum irisin levels than those with eGFR ≥ 60, indicating that irisin may also reflect renal filtration impairment.

Mechanistically, decreased irisin may not only be a marker but also a potential contributor to DN progression. Irisin is known to activate the PGC-1α pathway in muscle, enhancing mitochondrial biogenesis and reducing oxidative stress (28). Studies in diabetic mice have shown that irisin supplementation attenuates kidney inflammation and fibrosis by modulating pathways such as TGF-β signaling and oxidative/nitrosative stress reduction (29). In proximal tubular cells and ischemia-reperfusion injury models, irisin upregulates renal uncoupling protein 2 (UCP2) and glutathione peroxidase 4, mitigating mitochondrial damage and reactive oxygen species accumulation (18). These effects suggest that irisin exerts direct renoprotective actions beyond its systemic metabolic benefits.

Comparative literature further supports these findings. For example, Mageswari et al. (18) demonstrated that lower irisin levels were associated with higher albuminuria stages in T2DM patients. Similarly, Wang and Liu’s meta-analysis (13, 14) reported significantly decreased circulating irisin concentrations in T2DM, suggesting irisin’s potential role in mitigating insulin resistance and systemic inflammation. Formigari et al. (30) proposed that the renoprotective benefits of exercise may be mediated via the irisin/AMPK axis. Together, these studies reinforce the plausibility of irisin as both a marker and modulator of DN.

However, several limitations must be acknowledged. First, although funnel plots indicated low publication bias in most comparisons, asymmetry was observed in the microalbuminuria vs. macroalbuminuria subgroup, indicating possible small-study effects. Second, it is important to emphasize that substantial heterogeneity was observed across the included studies (I² > 80%), which may impact the robustness and interpretability of the pooled results. Although we addressed this by employing a random-effects model and conducting subgroup analyses (e.g., stratified by albuminuria stages and eGFR levels), residual heterogeneity remained. This heterogeneity likely arises from multiple sources, including differences in irisin detection methods (e.g., varying ELISA kits), biospecimen types (serum vs. plasma), population characteristics (e.g., geographic region, ethnicity, diabetes duration), and diagnostic criteria for diabetic nephropathy.

Such heterogeneity may limit the generalizability of our findings and complicate causal interpretations. In contrast, the meta-analysis conducted by Wang et al. (2021) (14), which evaluated serum irisin levels in patients with T2DM regardless of DN stage, reported relatively lower heterogeneity (I² ≈ 60%). This may be due to more consistent measurement techniques or broader inclusion criteria that did not differentiate DN severity. Our study, by focusing specifically on irisin differences across DN subgroups, captured more nuanced variability but at the cost of increased heterogeneity.

Nevertheless, sensitivity analyses demonstrated that the removal of individual studies did not substantially alter the overall effect sizes, indicating a degree of robustness. Future studies should aim to minimize heterogeneity by using standardized irisin assays, harmonized DN diagnostic criteria, and large-scale prospective designs to enhance the reliability and comparability of findings. While our findings suggest that decreased serum irisin levels are associated with the severity of DN, including both worsening albuminuria and reduced eGFR, the statement regarding its utility as a “biomarker” warrants clarification. Specifically, the current meta-analysis provides evidence of an association, but does not establish a diagnostic threshold or assess predictive performance metrics such as sensitivity, specificity, or ROC curves. Thus, while reduced irisin may reflect renal injury severity, its role as a diagnostic or prognostic biomarker remains exploratory rather than definitive.

None of the studies included in our meta-analysis reported a validated cut-off value for serum irisin that could distinguish between DN stages or predict progression. This contrasts with recent work by Berezina et al. (2024) (20), who evaluated the longitudinal trajectory of irisin in patients with asymptomatic heart failure and found that lower irisin levels independently predicted future kidney-related events. Importantly, their study proposed a threshold concentration below which the risk of renal deterioration significantly increased, thereby supporting the potential prognostic value of irisin.

To evaluate the clinical applicability of irisin in DN, future research should adopt a standardized approach to irisin measurement and seek to establish thresholds linked to renal endpoints. This will require prospective cohort studies that assess irisin levels over time in relation to DN onset and progression, ideally incorporating multivariate models adjusted for known risk factors (31–33). Until such thresholds are validated, the use of irisin as a clinical biomarker—whether diagnostic or prognostic—should be considered hypothesis-generating rather than actionable. In addition to our findings, a recent meta-analysis by Hou et al. (2023) (34) further supports the association between lower circulating irisin levels and diabetic complications. Their study evaluated the relationship between irisin levels and a range of diabetic microvascular complications, including diabetic nephropathy, retinopathy, and neuropathy, and found that irisin levels were consistently reduced in patients with these chronic complications compared to those without (15, 35). This broader association suggests that decreased irisin is not specific to diabetic nephropathy, but may reflect a general marker of microvascular disease burden in type 2 diabetes. While this reinforces the relevance of irisin in the pathophysiology of diabetic complications, it also highlights an important limitation: low irisin levels may not discriminate between different organ-specific complications. Therefore, irisin’s utility as a specific biomarker for DN remains uncertain and should be interpreted with caution (36).

To address these issues, future research should prioritize high-quality, multicenter prospective studies using standardized irisin quantification protocols. Stratification by ethnicity, glycemic control, physical activity level, and renal pathology stage will be crucial in elucidating the independent and causal role of irisin in DN. Additionally, mechanistic studies exploring the irisin-FNDC5 axis in human renal tissues and interventional trials assessing the effects of irisin-modulating therapies on renal outcomes will further clarify its utility as a diagnostic and therapeutic target.

### 4.1 Strengths of the study

This meta-analysis has several notable strengths. First, it is the most up-to-date and focused synthesis specifically evaluating the relationship between circulating irisin levels and DN, incorporating studies up to July 2025. Second, we performed detailed subgroup analyses based on albuminuria stages (normo-, micro-, macroalbuminuria) and renal function (eGFR ≥ or < 60 mL/min/1.73 m²), allowing for a more granular understanding of how irisin levels correlate with DN severity. Third, the inclusion of studies from both Western and Eastern populations, including Chinese-language databases, enhances the ethnic and geographic diversity of the evidence base. Finally, the use of sensitivity analyses and quality assessments (Jadad and NOS scales) adds rigor to the evaluation and increases the reliability of the conclusions drawn.

## 5 Conclusions

Through a comprehensive systematic review and meta-analysis, this study provides updated and robust evidence that serum irisin levels are significantly reduced in patients with DN, particularly those with microalbuminuria, macroalbuminuria, or decreased eGFR. These findings suggest that irisin may serve as a potential biomarker for early detection and disease stratification in DN. Compared to previous literature, our study offers a focused, stage-specific analysis of irisin’s relationship with renal function in type 2 diabetes, addressing a gap not fully explored in earlier meta-analyses.

Moreover, by incorporating both Western and Eastern studies, this work contributes a globally relevant synthesis and highlights the need for standardized measurement methods and validated threshold values to enhance irisin’s clinical utility. Despite the observed associations, the mechanistic underpinnings and specificity of irisin in DN versus other diabetic complications remain incompletely understood.

Therefore, future high-quality, multicenter randomized controlled trials with larger sample sizes are essential to validate irisin’s diagnostic and prognostic value and to clarify whether it plays an active pathophysiological role or merely reflects downstream effects of kidney dysfunction. This meta-analysis thus serves as a foundational reference for future biomarker research in diabetic kidney disease.

## Data Availability

The original contributions presented in the study are included in the article/Supplementary Material. Further inquiries can be directed to the corresponding author.
